# Un cas de dégénérescence astéroïde du vitré

**DOI:** 10.11604/pamj.2014.19.186.5421

**Published:** 2014-10-23

**Authors:** Fatima Zohra El Meriague, Rajae Daoudi

**Affiliations:** 1 Université Mohammed V Souissi, Service d'Ophtalmologie A de l'Hôpital des Spécialités, Centre Hospitalier Universitaire, Rabat, Maroc

**Keywords:** dégénérescence, astéroide, vitré, degeneration, asteroid, vitreous

## Image en medicine

Nous rapportons le cas d'un patient de 56 ans, sans antécédent pathologique, qui présente depuis, un cas d'une baisse de l'acuité visuelle progressive de l'oeil droit depuis un an associée à une photophobie. A l'examen, l'acuité visuelle au niveau de cet oeil est à 1/10. Le tonus est à 14 mmhg. L'examen du segment antérieur montre une cataracte nucléaire grade 1 et sous capsulaire postérieure. Au fond d'oeil, on est surpris par la découverte de particules blanchâtres et brillantes, de forme arrondie, de taille variable siégeant dans le vitré antérieur, mobiles aux mouvements du globe, typique d'une dégénérescence astéroïde du vitré. Le fond d'oeil est mal ou non visible. La baisse de l'acuité visuelle étant dûe principalement à la cataracte, le patient a bénéficié d'une chirurgie de la cataracte par technique de phacoémulsification. L'acuité visuelle du patient est remontée à 9/10 en post opératoire.

**Figure 1:**
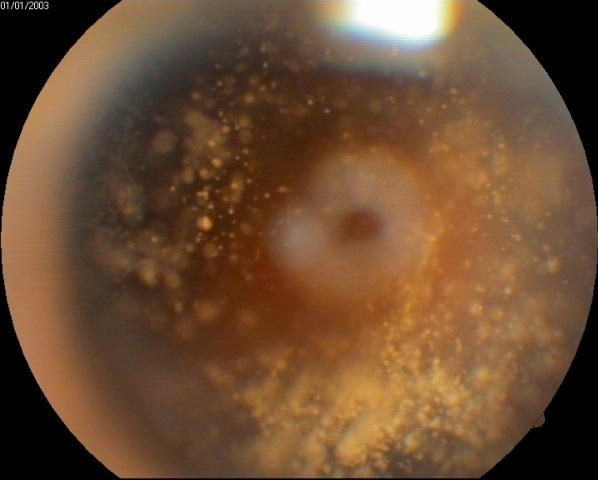
Fond d’œil montrant une dégénérescence astéroïde du vitré

